# Astrocytic induction of triglyceride lipolysis in AD‐associated pathology and neuroinflammation

**DOI:** 10.1002/alz.093056

**Published:** 2025-01-03

**Authors:** Till S Zimmer, Daniel Barnett, Adam L Orr, Anna G. Orr

**Affiliations:** ^1^ Weill Cornell Medicine, New York, NY USA; ^2^ Feil Family Brain and Mind Research Institute, Weill Cornell Medicine, New York, NY USA; ^3^ Appel Alzheimer’s Disease Research Institute, Weill Cornell Medicine, New York, NY USA; ^4^ Neuroscience Graduate Program, Weill Cornell Medicine, New York, NY USA; ^5^ Weill Cornell Medicine‐Rockefeller‐Sloan Kettering Tri‐Institutional MD‐PhD Program, New York, NY USA

## Abstract

**Background:**

Multiple AD risk genes are implicated in lipid metabolism, and plasma and brain lipid levels are altered in AD. Astrocytes are enriched in key lipid‐related factors and are likely contributors to altered lipid homeostasis in AD. We hypothesize that APP/Aβ‐related pathology and neuroimmune factors modulate astrocytic gene transcription that promote maladaptive changes in lipid pathways, including aberrant astrocytic production and release of lipids that could affect Aβ pathology and neuronal deficits.

**Method:**

To investigate the effects of APP/Aβ and neuroinflammation on astrocytic lipid metabolism, we used homozygous mutant APP knock‐in mice (APP^SAA^‐KI) that have progressive Aβ proteinopathy and gliosis, as well as mice injected systemically with lipopolysaccharide (LPS) to induce acute neuroinflammatory responses. Astrocyte‐specific analyses were performed *in vivo* by either FACS‐sorting astrocytes for downstream lipidomic profiling or by ribosome‐tagging astrocytes in mouse models (Ribotag) for astrocyte‐specific RNA sequencing. We also performed lipidomic, RNA, and protein‐based analyses in primary murine astrocytes stimulated with oligomeric Aβ (oAβ) or interleukin‐1 (IL‐1α/β).

**Result:**

Lipidomics in acutely sorted cortical astrocytes from APP^SAA^‐KI or LPS‐injected mice revealed decreased levels of triglycerides and cholesterol esters. In LPS‐injected mice, cortical astrocytes also had increased expression of enzymes involved in lipolysis, most notably adipose triglyceride lipase (*Atgl*), the principal triglyceride hydrolase. Similarly, primary astrocytes had increased levels of triglyceride lipolysis upon IL‐1α/β or oAβ stimulation, leading to increased liberation of free fatty acids (FFAs) in an NF‐κB and ERK1/2‐dependent manner. Astrocytic FFAs generated from triglyceride lipolysis were not used for energy production via β‐oxidation but instead utilized by astrocytes to produce prostaglandins (PGE_2_) and to facilitate secretion of extracellular vesicular bodies (EVB). Inhibiting ATGL decreased PGE_2_ production, revealing an essential role of astrocyte triglyceride lipolysis in neuroinflammatory processes.

**Conclusion:**

Our findings suggest that Aβ pathology and neuroinflammatory factors cause astrocytic triglyceride lipolysis, a process that liberates fatty acids to be utilized for various potentially pathogenic cascades in disease. We conclude that amyloid pathology and neuroimmune factors converge to alter astrocytic lipid‐related gene transcription and lipid‐based signaling.

